# Assessing the power of AI: a comparative evaluation of large language models in generating patient education materials in dentistry

**DOI:** 10.1038/s41405-025-00349-1

**Published:** 2025-06-18

**Authors:** Gowri Sivaramakrishnan, Maryam Almuqahwi, Sufyan Ansari, Mohammed Lubbad, Emad Alagamawy, Kannan Sridharan

**Affiliations:** 1 Bahrain Defence Force Royal Medical Services, Riffa, Bahrain; 2 Dental and Maxillofacial Center, Bahrain Defence Force Royal Medical Services, Riffa, Bahrain; 3 College of Medicine and Health Sciences, Arabian Gulf University, Manama, Bahrain

**Keywords:** Continuing professional development in dentistry, Dental patient management

## Abstract

**Background:**

This study evaluates the use of large language models (LLMs) in generating Patient Education Materials (PEMs) for dental scenarios, focusing on their reliability, readability, understandability, and actionability. The study aimed to assess the performance of four LLMs—ChatGPT-4.0, Claude 3.5 Sonnet, Gemini 1.5 Flash, and Llama 3.1–405b—in generating PEMs for four common dental scenarios.

**Methods:**

A comparative analysis was conducted where five independent dental professionals assessed the materials using the Patient Education Materials Assessment Tool (PEMAT) to evaluate understandability and actionability. Readability was measured with Flesch Reading Ease and Level scores, and inter-rater reliability was assessed using Fleiss’ Kappa.

**Results:**

Llama 3.1–405b demonstrated the highest inter-rater reliability (Fleiss’ Kappa: 0.78–0.89). ChatGPT-4.0 excelled in understandability, surpassing the PEMAT threshold of 70% in three of the four scenarios. Claude 3.5 Sonnet performed well in understandability for two scenarios but did not consistently meet the 70% threshold for actionability. ChatGPT-4.0 generated the longest responses, while Claude 3.5 Sonnet produced the shortest.

**Conclusions:**

ChatGPT-4.0 demonstrated superior understandability, while Llama 3.1–405b achieved the highest inter-rater reliability. The findings indicate that further refinement and human intervention is necessary for LLM-generated content to meet the standards of effective patient education.

## Introduction

Patient education materials (PEMs) play a crucial role in dental care, serving as an essential resource for educating individuals about their oral health, post-treatment care, and emergency management [1, 2]. These materials help bridge the gap between professional dental advice and patient understanding, ensuring that individuals can follow appropriate self-care practices at home [2]. In a dental setting, well-structured educational content can enhance patient compliance with treatment recommendations, reduce the risk of complications, and improve overall oral health outcomes [1]. Effective patient education materials should be clear, accurate, accessible, and actionable, enabling individuals to easily comprehend and apply the information provided [1, 3].

With advancements in artificial intelligence (AI), Large Language Models (LLMs) have emerged as a potential tool for generating PEMs efficiently and at scale [4, 5]. LLMs, such as Chat GPT 4.0, Claude 3.5 Sonnet, Gemini 1.5 Flash, and Llama 3.1–405b, are trained on vast amounts of textual data and can generate human-like responses to various prompts. These AI-powered models are increasingly being explored for their ability to simplify complex medical information, personalize health education, and improve accessibility for patients with diverse literacy levels [6–8]. While these models can generate fluent and coherent text, concerns remain regarding their accuracy, reliability, and readability when applied to healthcare contexts [9]. Given the high stakes of medical and dental information, it is critical to assess whether LLM-generated materials meet the standards of clarity, medical accuracy, and practical usability.

This study aims to evaluate the effectiveness of LLM-generated PEMs for common dental scenarios, focusing on their reliability, readability, and actionability. By assessing materials generated for four key dental situations, this study sought to determine whether AI-generated content aligns with the principles of effective health communication. The findings of this study provide insights into the strengths and limitations of LLMs in generating PEMs, helping to inform future applications of AI in dental communication and patient care.

## Methodology

### Study design and selection of large language models

**Fig. 1:**
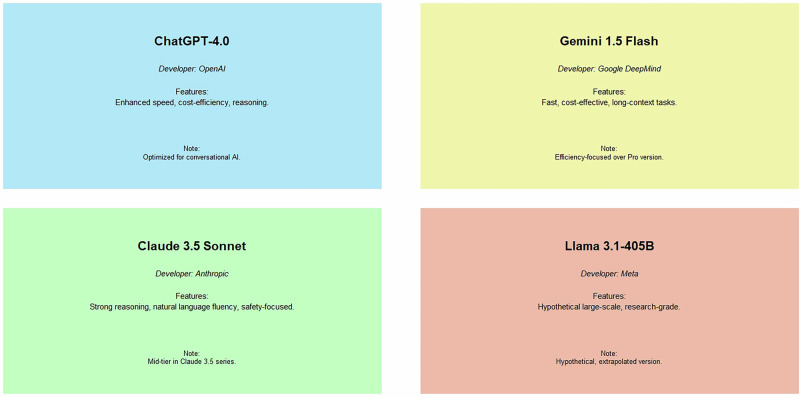
Large language models (LLMs) evaluated in this study. The figure summarizes key attributes of four LLMs analyzed. Developer information and feature highlights are provided for each model.

This study used a comparative analytical design to evaluate the reliability, readability, and actionability of patient education materials generated by four LLMs: ChatGPT-4.0, Claude 3.5 Sonnet, Gemini 1.5 Flash, Llama 3.1–405B. Figure 1 summarizes the key features of these LLMs. The selection of these models ensured a balanced evaluation of both proprietary and open-source LLM’s in dental health communication. Due to the nature of the study, ethical approval was not sought. However, adherence to the latest Declaration of Helsinki guidelines was maintained.

Each model was prompted to generate patient education handouts for four specific dental scenarios:

- Post-operative instructions following a tooth extraction
- Immediate steps for managing an avulsed tooth
- Proper daily tooth brushing technique for optimal oral hygiene
- Self-examination for oral cancer screening

For consistency, the prompts were carefully structured to ensure that each LLM received identical instructions without additional context or examples. The prompts were designed to be succinct and clear, ensuring that they were easily understandable and replicable by anyone using the same set of instructions. This approach was aimed at minimizing any biases or variations that might arise from differing interpretations or added context. By using straightforward, concise prompts, the study ensured that each model’s performance was based solely on the input provided, allowing for an unbiased and uniform evaluation of the generated materials. The generated materials were then assessed using multiple standardized evaluation metrics.

### Assessment of readability, actionability and understandability

The Patient Education Materials Assessment Tool (PEMAT) [3] was used to assess the understandability and actionability of the materials generated by each LLM. Five independent dental professionals, rated each of the four generated materials using the PEMAT criteria. The ratings focused on evaluating how easy the content was to understand (understandability) and how clearly patients could identify and apply the actions or steps recommended (actionability). For each material, mean scores were calculated for both understandability and actionability, allowing for a comprehensive evaluation of each LLM’s output. For understandability, a score of 70% or above indicates that the material is understandable for most patients. For accountability, a score of 70% or higher is considered good, meaning the material clearly outlines actions that are easy to follow [3].

In addition to the PEMAT, other readability scores, such as the Flesch Reading Ease and Reading Level scores, were also calculated to evaluate the accessibility of the materials in terms of their linguistic complexity. These scores were calculated using the online calculators that are freely available. These scores allowed for further comparison between the LLMs, focusing on the ease of reading and the suitability of the language used for various patient populations.

### Inter-rater reliability

To assess the consistency and agreement among the five raters, Fleiss’ Kappa was used to measure inter-rater reliability. The level of agreement was categorized according to standard Fleiss’ Kappa interpretation:

- No agreement (≤0)
- Slight (0.01–0.20)
- Fair (0.21–0.40)
- Moderate (0.41–0.60)
- Substantial (0.61–0.80)
- Almost perfect agreement (0.81–1.00)

## Results

All LLMs provided responses to each of the scenarios outlined, and these responses are presented in Supplementary File 1.

### Inter-rater reliability

**Fig. 2:**
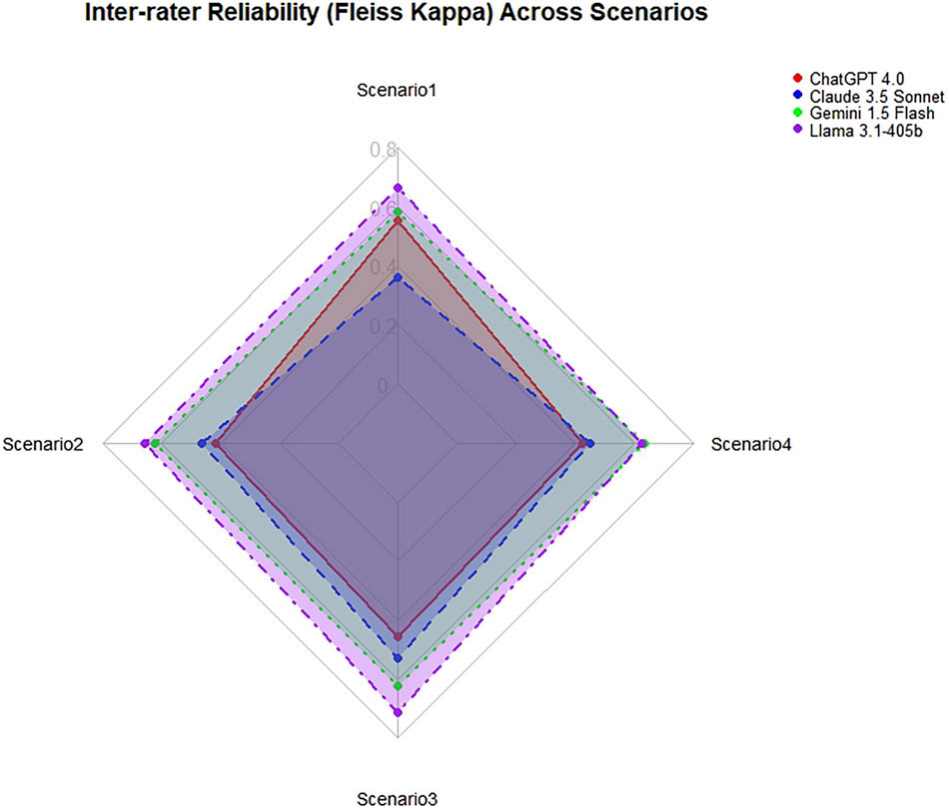
Inter-rater reliability across scenarios for each LLM. Radar chart comparing Fleiss Kappa scores of four large language models (LLMs) across four scenarios. Each axis represents a scenario, with Fleiss Kappa values plotted radially from the center. Model performance is shown as distinct geometric lines. Higher values toward the outer edges indicate stronger inter-rater agreement.

Llama 3.1–405b demonstrated the highest level of inter-rater reliability, with Fleiss’ Kappa values ranging from 0.78 to 0.89, indicating almost perfect agreement among the raters for both understandability and actionability across the five evaluations. Chat GPT 4.0 displayed substantial agreement, particularly in the ratings for actionability (*κ* = 0.69), but showed moderate agreement in the other areas, with Fleiss’ Kappa values ranging from 0.52 to 0.57. Claude 3.5 Sonnet exhibited moderate inter-rater reliability, with Fleiss’ Kappa values ranging from 0.45 to 0.66. Gemini 1.5 Flash demonstrated reasonable consistency with Fleiss’ Kappa values ranging from 0.73 to 0.79, reflecting a consistent level of agreement, though not as strong as Llama 3.1–405b. These findings suggest that while all models demonstrated acceptable inter-rater reliability, Llama 3.1–405b emerged as the most reliable model, particularly in generating materials with high consistency across raters (Supplementary File 2). The radar plot is presented in Fig. 2.

### Understandability and actionability

#### Scenario 1- post-operative instructions following tooth extraction

**Fig. 3:**
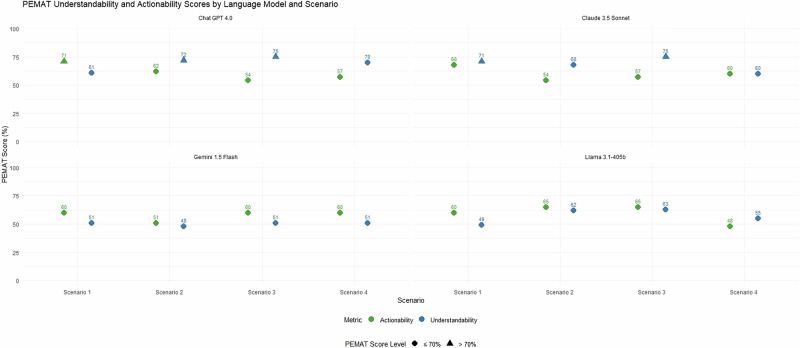
PEMAT understandability and actionability scores across scenarios for each LLM. Line graphs comparing Patient Education Materials Assessment Tool (PEMAT) scores for four large language models (LLMs) across four scenarios. Scenarios are plotted along the X-axis, with PEMAT percentage scores (0–100%) on the Y-axis.

The understandability scores for this scenario varied across the models. Chat GPT 4.0 scored 61% for understandability in Scenario 1, indicating moderate clarity, while Llama 3.1–405b scored 49%, suggesting that the content generated by this model may have been more complex and less clear. Chat GPT 4.0 had the highest actionability score at 71% in Scenario 1, indicating a high level of practical guidance. Llama 3.1–405b and Gemini 1.5. Flash scored lower at 60%, suggesting that the instructions were less actionable and may have lacked specific details for patients to follow effectively (Fig. 3).

#### Scenario 2- immediate steps for managing an avulsed tooth

Chat GPT 4.0 scored 72% for understandability indicating clear instructions for handling the situation. Gemini 1.5 Flash scored the lowest at 48%, reflecting potentially more technical language or unclear phrasing. Llama 3.1–405b led in actionability with a score of 65%, demonstrating practical guidance for handling the avulsed tooth and seeking dental care. The other models, such as Claude 3.5 Sonnet, Gemini 1.5 Flash and Chat GPT, had relatively lower actionability scores (54%, 51% and 62%, respectively), indicating that the instructions were less detailed or actionable (Fig. 3).

#### Scenario 3- proper daily tooth brushing technique

Gemini 1.5 Flash scored relatively low for understandability at 51%. Chat GPT 4.0 and Claude 3.5 Sonnet scored higher (75%), reflecting more accessible content. Llama 3.1–405b scored the highest for actionability at 65%, indicating that the material included clear, step-by-step instructions (Fig. 3).

#### Scenario 4- self-examination for oral cancer screening

Chat GPT achieved the highest understandability scores (70%) indicating clearer communication of the steps involved in oral cancer self-examination. Claude 3.5 Sonnet and Gemini 1.5 Flash led in actionability, with a score of 60%, indicating actionable guidance on how to perform the self-examination and seek professional care when necessary (Fig. 3). Among the evaluated language models, only Chat GPT 4.0 and Claude 3.5 Sonnet achieved understandability scores above 70% in certain scenarios. Chat GPT 4.0 demonstrated strong performance in understandability, surpassing the 70% threshold in Scenario 2, 3 and 4. Additionally, it was the only model to achieve an actionability score above 70% (Scenario 1). Claude 3.5 Sonnet also performed well in understandability, exceeding 70% in Scenario 1 and 3. However, none of the models consistently met the 70% benchmark for both understandability and actionability across all scenarios, highlighting the variability in performance depending on the context.

### Readability

Chat GPT 4.0 showed a moderate readability range, with scores between 52.2 and 69.9 across all scenarios. This suggests that its responses were readable at an 8th or 9th-grade level. Claude 3.5 Sonnet displayed variability in readability, with the most difficult text in Scenario 1 (Flesch score of 57.4), while Scenario 2 was easier to read (74.0). Scenarios 3 and 4, however, were harder to read (41.7 and 49.8), which could present challenges for a general audience. Gemini 1.5 Flash produced text that was relatively difficult to read across all scenarios, indicating a high reading difficulty level (10th to 12th-grade level). Llama 3.1–405b exhibited more variation, with Scenario 3 showing the best readability (76.8, equivalent to a 7th-grade level), while other scenarios scored lower (Table 1).

**Table 1:** Characteristics of responses received.

Source	Scenario	Flesch reading ease score	Reading level	Average words per sentence	Total sentences	Total words
Chat GPT 4.o	1	52.2	10th to 12th grade (Fairly difficult to read)	10.7	47	505
	2	67.6	8th & 9th grade (Plain English)	12.2	43	524
	3	60.8	8th & 9th grade (Plain English)	10.5	57	599
	4	69.9	8th & 9th grade (Plain English)	9.9	66	655
Claude 3.5 Sonnet	1	57.4	10th to 12th grade (Fairly difficult to read)	5.5	31	171
	2	74	7th grade (Fairly easy to read)	5.8	37	213
	3	41.7	College (Difficult to read)	4.3	51	218
	4	49.8	College (Difficult to read)	4.7	58	274
Gemini 1.5 Flash	1	54.2	10th to 12th grade (Fairly difficult to read)	8.7	63	551
	2	54.9	10th to 12th grade (Fairly difficult to read)	8	57	456
	3	52.6	10th to 12th grade (Fairly difficult to read)	10.3	51	525
	4	52.3	10th to 12th grade (Fairly difficult to read)	10.6	53	563
Llama 3.1–405b	1	53.8	10th to 12th grade (Fairly difficult to read)	9.1	40	362
	2	62.1	8th & 9th grade (Plain English)	9.2	39	360
	3	76.8	7th grade (Fairly easy to read)	11.4	39	443
	4	69.6	8th & 9th grade (Plain English)	9.9	47	467

### Word count and sentence structure

The length of responses and sentence structure varied across the models. Chat GPT 4.0 generated the longest responses, averaging 505–655 words per scenario. Its sentence length ranged from 9.9 to 12.2 words, indicating that it tended to produce more detailed responses with relatively long sentences. Claude 3.5 Sonnet generated the shortest responses, averaging between 456 and 551 words. Its sentence structure was more concise, with an average of 4.3–5.8 words per sentence, indicating a more direct and to-the-point approach in response generation. Gemini 1.5 Flash produced responses of similar length to Claude 3.5 Sonnet, with an average of 470 words. Llama 3.1–405b produced responses averaging between 362 and 467 words, with sentence lengths ranging from 9.1 to 11.4 words (Table 1).

## Discussion

The aim of this study was to evaluate the performance of four LLM’s—Chat GPT 4.0, Claude 3.5 Sonnet, Gemini 1.5 Flash, and Llama 3.1–405b—in generating dentistry-related content across four different scenarios. The focus was on assessing inter-rater reliability, understandability, actionability, readability, and response characteristics. The findings indicate notable variations in model performance based on these criteria. Llama 3.1–405b demonstrated superior inter-rater reliability, indicating consistent ratings across raters, but it performed less well in understandability and accountability compared to Chat GPT 4.0.

Based on the recommended 6th to 8th grade reading level by both the American Medical Association (AMA) and the National Institutes of Health (NIH) [1, 10, 11], this range is recommended because many patients have reading skills at or below this level, and health materials above this threshold risk being too complex, potentially limiting comprehension and effective self-care. The results of this analysis showed a mixed performance across the LLMs. Llama 3.1–405b and Claude 3.5 Sonnet were the closest to meeting this recommendation, with one scenario each falling within the 7th to 8th grade range. However, Chat GPT 4.0 and Gemini 1.5 Flash tended to produce content at a higher grade level, for all scenarios, which may make the material more challenging for patients to understand. Readability formulas like Flesch-Kincaid provide quantitative estimates but may not fully capture complexity due to medical jargon or sentence structure. This highlights the importance of human oversight to ensure language is appropriately simple and clear for diverse patient populations. While these models performed well in many aspects, none of them consistently hit the ideal 6th grade level, highlighting the need for human intervention to simplify the content to align with the recommended readability levels.

Our findings highlight notable differences in readability, word count, and sentence structure across the LLMs evaluated. Interestingly, these factors can be influenced by how the prompts are framed. For example, explicitly instructing the models to “use simple and easy words so that a sixth grader can understand” or “limit responses to 100 words” may improve readability and conciseness. Such strategies are valuable for tailoring LLMs outputs to different audiences or scenarios, especially in health communication or patient education contexts. Future work could explore systematically how prompt modifications affect readability and length across various models and scenarios.

Patient education materials should be clear, concise, and easily understandable to ensure effective communication [12]. Key features include simple, non-technical language that is accessible to a wide range of literacy levels, along with a logical structure that guides the reader through the content [13]. Visual aids, such as diagrams, infographics, or images, are crucial in enhancing understanding and providing clarity for complex medical concepts [14]. Actionable steps or instructions should be prominently highlighted to help patients follow through with care recommendations. Furthermore, the material should be culturally sensitive and tailored to the patient’s specific needs, ensuring that it resonates with their background and health conditions [15, 16]. It should also include clear contact information for further questions or assistance, fostering patient engagement and empowerment. Lastly, materials should be visually appealing, with a clean layout and ample white space to make it easy for patients to navigate and focus on important information. The responses received from all four models included in this study did not include any images, infographics, or visual representations primarily because these models are designed to generate and process text-based content only. While they excel at providing written responses, they are not inherently equipped to produce or interpret visual elements like images or diagrams [17]. However, it is important to note that ChatGPT 4.0 does have the capability to generate images in some contexts, depending on the platform and settings used. Despite this, the models remain focused on generating human-readable text for a variety of applications, including healthcare communication, but generally lack the integration of image creation or editing functionalities [17–19]. As a result, their output is limited to textual information, making it necessary for human intervention to add visual aids, such as images or infographics, during the final stages of content development, especially for PEMs where visual aids play a crucial role in improving comprehension.

In addition to images, LLMs cannot offer personalized content tailored to an individual’s specific health condition, demographic, or preferences, as they rely on general inputs. To overcome the general-purpose nature of these models and improve their domain specificity, recent efforts have focused on fine-tuning LLMs using approaches such as Retrieval Augmented Generation (RAG). RAG combines LLMs with external knowledge retrieval, allowing models to access up-to-date and specialized information relevant to a user’s query. This method can enhance the accuracy and contextual relevance of generated content in healthcare settings. Batool et al. [20]. demonstrated the use of an embedded GPT model tailored for post-operative dental care, showing improved performance compared to standard ChatGPT. Similarly, Umer et al. [21]. applied RAG-enhanced LLM techniques to transform educational journal clubs, addressing specific learning challenges. Incorporating such domain-adapted models may bridge the gap between generalist LLM outputs and the need for precise, personalized patient education materials. They also lack the ability to generate real-time updates or access live data, meaning that the content may not reflect the most current clinical guidelines or patient outcomes. These models also do not provide clinical decision support, patient-specific instructions, or ensure compliance with local healthcare regulations, making human oversight necessary. Furthermore, LLMs cannot replicate the human element of empathy, which is essential for reassuring patients, nor do they always account for cultural sensitivities or provide reliable citations [22, 23]. As a result, while LLMs can generate informative content, they are not fully equipped to produce dynamic, personalized, and compliant patient information materials without human intervention.

One limitation of the current study relates to the simplicity of the prompts provided to the LLMs. Although identical base prompts were used for all models in our study to maintain consistency and minimize variability due to prompt design, these prompts were intentionally kept basic. It is well-established in the literature that the quality of LLM outputs depends heavily on the quality and specificity of the prompts given [24–26]. More complex or detailed prompts could potentially elicit more accurate or nuanced responses from the models [27]. However, we deliberately chose simple prompts to simulate typical real-world scenarios where users may not craft elaborate instructions. This approach reflects practical conditions under which PEMs might be generated by users with limited expertise in prompt engineering. Future research could explore how varying prompt complexity impacts the quality of generated health communication materials.

This study evaluated LLM performance using only four dental scenarios. While these scenarios were chosen for their clinical relevance and diversity—covering preventive care, emergency management, routine post-treatment instructions, and early detection—they represent only a subset of the broad range of patient education needs in dentistry. Consequently, the findings may have limited generalizability to other dental topics or more complex clinical situations. Future research should include a wider variety of scenarios to better assess the comprehensive capabilities of LLMs in dental patient education.

In conclusion, while LLMs demonstrate promising capabilities in generating patient education materials, their current limitations underscore the critical need for human oversight and intervention. Although these models excel at producing coherent text-based content, they generally lack the ability to create visual aids, tailor information to individual patient characteristics, or integrate real-time clinical data. Additionally, LLMs cannot fully replicate essential human qualities such as empathy and cultural sensitivity, which are crucial for effective healthcare communication. Recent advancements, including fine-tuning approaches like RAG, offer pathways to enhance model specificity and relevance in healthcare domains. However, even with these improvements, LLM-generated content should be considered as a supportive tool for healthcare professionals rather than a standalone solution. Ensuring optimal patient understanding and engagement requires continued refinement of these models combined with active human involvement to address their current shortcomings.

## Supplementary information

Supplementary Information

## Data Availability

The datasets used and/or analyzed during the current study are available from the corresponding author on reasonable request.

## References

[1] Alexander RE. Readability of published dental educational materials. J Am Dent Assoc. 2000;131:937–42. 10.14219/jada.archive.2000.0312. 10916332 10.14219/jada.archive.2000.0312

[2] Ho JCY, Chai HH, Lo ECM, Huang MZ, Chu CH. Strategies for effective dentist-patient communication: a literature review. Patient Preference Adherence. 2024;18:1385–94. 10.2147/PPA.S465221. 38974679 10.2147/PPA.S465221 PMC11225999

[3] Shoemaker SJ, Wolf MS, Brach C. Development of the patient education materials assessment tool (PEMAT): a new measure of understandability and actionability for print and audiovisual patient information. Patient Educ Counsel. 2014;96:395–403. 10.1016/j.pec.2014.05.027. 10.1016/j.pec.2014.05.027 PMC5085258 24973195

[4] Chiesa-Estomba CM, Lechien JR, Vaira LA, Brunet A, Cammaroto G, Mayo-Yanez M, et al. Exploring the potential of Chat-GPT as a supportive tool for sialendoscopy clinical decision making and patient information support. Eur Arch Otorhinolaryngol. 2024;281:2081–6. 10.1007/s00405-023-08104-8. 37405455 10.1007/s00405-023-08104-8

[5] Shamil E, Jaafar M, Fan KS, Ko TK, Schuster-Bruce J, Eynon-Lewis N, et al. The use of large language models such as ChatGPT on delivering patient information relating to surgery. Facial Plastic Surg. 2024. 10.1055/a-2413-3529. 10.1055/a-2413-3529 39260419

[6] Patil S, Shankar H. Transforming healthcare: harnessing the power of AI in the modern era. Int J Multidiscip Sci Arts. 2023;2:60–70.

[7] Alowais SA, Alghamdi SS, Alsuhebany N, Alqahtani T, Alshaya AI, Almohareb SN, et al. Revolutionizing healthcare: the role of artificial intelligence in clinical practice. BMC Med Educ. 2023;23:689. 10.1186/s12909-023-04698-z. 37740191 10.1186/s12909-023-04698-z PMC10517477

[8] Thacharodi A, Hassan S, Vithlani A, Ahmed T, Kavish S, Geli Blacknell NM, et al. Revolutionizing healthcare and medicine: the impact of modern technologies for a healthier future—a comprehensive review. Health Care Sci. 2024;3:329–49. 10.1002/hcs2.115. 39479277 10.1002/hcs2.115 PMC11520245

[9] Bélisle-Pipon J-C. Why we need to be careful with LLMs in medicine. Front Med. 2024;11. 10.3389/fmed.2024.1495582. 10.3389/fmed.2024.1495582 PMC11652181 39697212

[10] National Institutes of Health. MedlinePlus: how to write easy to read health materials. Available at https://cir.nii.ac.jp/crid/1571417126226435840. Accessed 2025.

[11] Centers of Disease Control, Prevention. Simply put: a guide for creating easy-to-understand materials. Atlanta, GA: Centers for Disease Control and Prevention.

[12] Hansberry DR, Agarwal N, Shah R, Schmitt PJ, Baredes S, Setzen M, et al. Analysis of the readability of patient education materials from surgical subspecialties. Laryngoscope. 2014;124:405–12. 10.1002/lary.24261. 23775508 10.1002/lary.24261

[13] Hoffmann T, Worrall L. Designing effective written health education materials: considerations for health professionals. Disabil Rehabilit. 2004;26:1166–73. 10.1080/09638280410001724816. 10.1080/09638280410001724816 15371031

[14] Cohen SM, Baimas-George M, Ponce C, Chen N, Bain PA, Ganske IM, et al. Is a picture worth a thousand words? A scoping review of the impact of visual aids on patients undergoing surgery. J Surg Educ. 2024;81:1276–92. 10.1016/j.jsurg.2024.06.002. 38955659 10.1016/j.jsurg.2024.06.002

[15] Ho EY, Tran H, Chesla CA. Assessing the cultural in culturally sensitive printed patient-education materials for Chinese Americans with type 2 diabetes. Health Commun. 2015;30:39–49. 10.1080/10410236.2013.835216. 24446839 10.1080/10410236.2013.835216 PMC4105327

[16] Haynes D, Hughes KD, Okafor A. PEARL: a guide for developing community-engaging and culturally-sensitive education materials. J Immigr Minority Health. 2023;25:666–73. 10.1007/s10903-022-01418-5. 10.1007/s10903-022-01418-5 PMC9584241 36266493

[17] Thirunavukarasu AJ, Ting DSJ, Elangovan K, Gutierrez L, Tan TF, Ting DSW. Large language models in medicine. Nat Med. 2023;29:1930–40. 10.1038/s41591-023-02448-8. 37460753 10.1038/s41591-023-02448-8

[18] Safranek CW, Sidamon-Eristoff AE, Gilson A, Chartash D. The Role of large language models in medical education: applications and implications. JMIR Med Educ. 2023;9:e50945 10.2196/50945. 37578830 10.2196/50945 PMC10463084

[19] Deng J, Zubair A, Park Y-J. Limitations of large language models in medical applications. Postgrad Med J. 2023;99:1298–9. 10.1093/postmj/qgad069. 37624143 10.1093/postmj/qgad069

[20] Batool I, Naved N, Kazmi SMR, Umer F. Leveraging Large Language Models in the delivery of post-operative dental care: a comparison between an embedded GPT model and ChatGPT. BDJ Open. 2024;10:1–7. 10.1038/s41405-024-00226-3. 38866751 10.1038/s41405-024-00226-3 PMC11169374

[21] Umer F, Naved N, Naseem A, Mansoor A, Kazmi SMR. Transforming education: tackling the two sigma problem with AI in journal clubs – a proof of concept. BDJ Open. 2025;11:1–5. 10.1038/s41405-025-00338-4. 40341404 10.1038/s41405-025-00338-4 PMC12062218

[22] Wang D, Zhang S. Large language models in medical and healthcare fields: applications, advances, and challenges. Artif Intell Rev. 2024;57:299 10.1007/s10462-024-10921-0.

[23] Laskar MTR, Alqahtani S, Bari MS, Rahman M, Khan MAM, Khan H, et al. A systematic survey and critical review on evaluating large language models: challenges, limitations, and recommendations. In: Al-Onaizan Y, Bansal M, Chen Y-N, editors. In: Proceedings of the 2024 conference on empirical methods in natural language processing. Miami, Florida, USA: Association for Computational Linguistics; 2024. p. 13785–816.

[24] Jacobsen LJ, Weber KE. The promises and pitfalls of large language models as feedback providers: a study of prompt engineering and the quality of AI-driven feedback. MDPI. 2025;6:35.

[25] Mao W, Wu J, Chen W, Gao C, Wang X, He X. Reinforced prompt personalization for recommendation with large language models. ACM Trans Inf Syst. 2025;43:1–27. 10.1145/3716320.

[26] Zhou H, Hu C, Yuan D, Yuan Y, Wu D, Chen X, et al. Large language models for wireless networks: an overview from the prompt engineering perspective. In: IEEE Wireless Communications. IEEE; 2025;1-9. 10.1109/MWC.001.2400384.

[27] Maaz S, Palaganas JC, Palaganas G, Bajwa M. A guide to prompt design: foundations and applications for healthcare simulationists. Front Media SA. 2025:11. 10.3389/fmed.2024.1504532 PMC11841430 39980724

