# Supplementary material for: Assessing the power of AI: a comparative evaluation of large language models in generating patient education materials in dentistry
Source: BDJ Open. 2025 Jun 18;11:59. doi: 10.1038/s41405-025-00349-1 (PMC12177049; doi:10.1038/s41405-025-00349-1)
Supplement: Supplementary file 1 — Supplementary Information [file 41405_2025_349_MOESM1_ESM.docx]

**Supplementary file 1- Responses received from the LLM’s included in our study**

**1.Scenario 1:** Provide a clear, printable patient education handout explaining post-operative instructions for patients following a tooth extraction in a dental clinic. Include specific guidance on managing pain, reducing swelling, dietary restrictions, and steps to promote healing, as well as signs of complications that require immediate follow-up with the dentist.

**1.1. Chat GPT:**

Post-Tooth Extraction Care Instructions

Following your tooth extraction, it is important to follow these instructions carefully to promote healing, minimize discomfort, and prevent complications. Below is a list of recommendations to help you through your recovery process.

Pain Management

Some discomfort is normal after a tooth extraction. Here's how you can manage it:

• Take pain medications as prescribed by your dentist. Over-the-counter pain relievers like ibuprofen (Advil) or acetaminophen (Tylenol) may also be recommended.

• Start taking pain medication before the local anesthetic completely wears off.

• If antibiotics were prescribed, take them as directed to prevent infection. Complete the entire course even if you feel better.

• Avoid aspirin for pain relief as it can increase bleeding.

Swelling and Bleeding

Mild swelling and bleeding are normal after an extraction. Here's how you can manage them:

• To minimize swelling, apply an ice pack to the outside of your cheek near the extraction site for 10-20 minutes at a time during the first 24 hours.

• Keep your head elevated when resting to reduce swelling and bleeding.

• If bleeding continues, bite down gently on a piece of gauze for 30-45 minutes. If bleeding persists after several hours, contact your dentist.

• Avoid rinsing or spitting forcefully for the first 24 hours as it may dislodge the blood clot formed at the extraction site.

Dietary Restrictions

For the first few days, follow these dietary guidelines to protect the extraction site:

• Stick to soft foods such as yogurt, mashed potatoes, applesauce, or smoothies for the first 24-48 hours.

• Avoid hot liquids and foods, as heat can increase swelling and cause discomfort.

• Do not use a straw or chew hard foods, as sucking or excessive chewing can dislodge the blood clot and delay healing.

• Gradually return to your normal diet as you feel comfortable, typically after a few days.

Promoting Healing

Follow these steps to promote healing and avoid complications:

• Maintain good oral hygiene, but avoid brushing directly over the extraction site for the first 24 hours.

• After 24 hours, gently rinse your mouth with warm salt water (1/2 teaspoon salt in 8 oz. of water) several times a day to reduce bacteria and promote healing.

• Avoid smoking and alcohol for at least 72 hours, as they can interfere with healing and increase the risk of complications like dry socket.

• Avoid strenuous physical activity for the first 24-48 hours, as it can increase bleeding and delay healing.

Signs of Complications

If you experience any of the following, contact your dentist immediately:

• Severe pain that does not improve after a few days or worsens.

• Excessive bleeding that does not stop after applying pressure with gauze.

• Swelling that worsens or lasts longer than three days.

• Fever or chills, which may indicate an infection.

• Foul taste or odor from the extraction site.

• Difficulty swallowing or breathing.

If you have any concerns or experience any complications, please contact your dental office:

Phone: [Insert Phone Number]

Email: [Insert Email Address]

Office Hours: [Insert Hours]

Thank you for following these instructions. We wish you a smooth and quick recovery!

**1.2. Claude 3.5. Sonnet**

Post-Tooth Extraction Care Instructions

First 24 Hours

• Keep the gauze pad in place with firm pressure for 30-60 minutes

• Avoid rinsing, spitting, or using a straw

• No smoking or alcohol

• Rest with your head elevated

• Apply ice packs: 20 minutes on, 20 minutes off

Managing Pain and Swelling

• Take prescribed medications as directed

• Continue ice packs for the first 24-48 hours

• Switch to warm compresses after 48 hours if needed

Diet Recommendations

First 24-48 Hours:

• Soft, cool foods only (yogurt, pudding, smoothies)

• Lukewarm soups

• Avoid hot foods and beverages

• Do not use straws

Oral Hygiene

• After 24 hours, gentle warm salt water rinses (½ teaspoon salt in 8 oz warm water)

• Brush teeth gently, avoiding extraction site

• Continue normal oral hygiene in other areas

Warning Signs - Call Your Dentist If:

• Severe pain not relieved by medication after 48 hours

• Heavy bleeding that won't stop

• Fever over 101°F (38.3°C)

• Swelling that worsens after 48-72 hours

• Difficulty breathing or swallowing

Clinic Contact Information:

Phone: (XXX) XXX-XXXX

Emergency After Hours: (XXX) XXX-XXXX

**1.3. Gemini 1.5. Flash**

Post-Operative Instructions Following Tooth Extraction

Patient Name: _________________________ Date of Extraction: _________________________

Tooth Extracted: _________________________

This handout provides important instructions to ensure a smooth recovery after your tooth extraction. Please read carefully and follow these guidelines closely.

I. Controlling Bleeding and Swelling:

Immediately after the extraction: Bite firmly on the gauze pad placed over the extraction site for at least 30-45 minutes. If bleeding continues, replace the gauze with a fresh pad and bite firmly for another 30 minutes. You may need to repeat this process for several hours.

After the initial bleeding stops: Avoid rinsing, spitting, or touching the extraction site with your tongue for at least 24 hours. This can dislodge the blood clot, leading to a "dry socket" (a painful complication).

Swelling: Swelling is normal and usually peaks 24-48 hours after the extraction. Apply a cold compress (ice wrapped in a thin cloth) to your cheek for 15-20 minutes at a time, several times a day, for the first 24-48 hours. This helps reduce swelling and pain. After 48 hours, you can switch to warm compresses to promote healing.

II. Pain Management:

Pain medication: Take prescribed pain medication as directed by your dentist. Do not exceed the recommended dosage. Over-the-counter pain relievers like ibuprofen (Advil, Motrin) or acetaminophen (Tylenol) can also help manage pain. Always follow the label instructions.

Pain level: If your pain becomes unmanageable despite taking medication, contact your dentist immediately.

III. Dietary Restrictions:

First 24 hours: Avoid eating or drinking anything hot or spicy for at least the first 24 hours. Stick to soft, cool foods like yogurt, applesauce, pudding, or broth. Avoid using straws, as the suction can dislodge the blood clot.

Following 24 hours: Gradually introduce softer foods back into your diet. Avoid hard, crunchy, or chewy foods for at least a week, or until the extraction site has healed sufficiently. Avoid foods that could get lodged in the extraction site.

IV. Oral Hygiene:

Gentle brushing: Begin brushing your teeth gently the day after the extraction, avoiding the extraction site. Focus on brushing the other teeth thoroughly.

Rinsing: After 24 hours, you can gently rinse your mouth with warm salt water (1/2 teaspoon salt in 8 ounces of warm water) several times a day. This helps keep the area clean and can promote healing. Avoid vigorous rinsing.

V. Promoting Healing:

Rest: Get plenty of rest to allow your body to heal.

Hydration: Drink plenty of fluids to stay hydrated.

Follow-up: Attend your scheduled follow-up appointment with your dentist.

VI. Signs of Complications – Contact your dentist immediately if you experience:

Excessive bleeding: Bleeding that soaks through gauze pads consistently after several hours.

Severe pain: Pain that is not controlled by prescribed medication.

Increased swelling: Swelling that increases significantly after 48 hours.

Fever: A temperature of 101°F (38.3°C) or higher.

Pus or foul odor: Noticeable pus or a bad odor coming from the extraction site.

Numbness that persists: Numbness that lasts longer than expected.

Dry socket: Severe pain, usually starting 2-3 days after the extraction, often accompanied by a visible empty socket.

VII. Contact Information:

[Dentist's Name]

[Dentist's Phone Number]

[Dentist's Address]

[Dentist's Emergency Number (if applicable)]

Disclaimer: This handout provides general post-operative instructions. Individual experiences may vary. Always follow your dentist's specific instructions. This information is not a substitute for professional medical advice.

**4.1. Llama 3.1-405b**

Post-Operative Instructions for Tooth Extraction

Introduction

Congratulations on taking the first step towards a healthier smile! Following a tooth extraction, it's essential to follow these instructions carefully to ensure a smooth and comfortable recovery.

Managing Pain

Take the prescribed pain medication as directed by your dentist.

For mild pain, you can take over-the-counter pain relievers such as ibuprofen (Advil, Motrin) or acetaminophen (Tylenol).

Apply an ice pack to the affected area for 15-20 minutes, 3-4 times a day, to reduce pain and swelling.

Reducing Swelling

Apply an ice pack to the affected area for 15-20 minutes, 3-4 times a day, to reduce swelling.

Elevate your head while sleeping to reduce swelling.

Avoid strenuous activities, bending, or heavy lifting for 24 hours.

Dietary Restrictions

Eat soft, bland foods such as yogurt, scrambled eggs, mashed potatoes, and soup for 24 hours.

Avoid hot, spicy, or acidic foods and drinks.

Avoid chewing on the extraction site until the stitches are removed or the site is fully healed.

Promoting Healing

Bite on the gauze for 30-45 minutes after the extraction to control bleeding.

Replace the gauze with a new one if it becomes soaked with blood.

Avoid smoking, tobacco products, and vaping for at least 24 hours.

Avoid drinking through a straw or spitting for 24 hours.

Keep the extraction site clean by rinsing with warm salt water (1/2 teaspoon of salt in 8 ounces of water) 2-3 times a day.

Signs of Complications

Excessive bleeding that doesn't stop after 30 minutes of biting on gauze

Severe pain that doesn't respond to pain medication

Swelling, redness, or pus around the extraction site

Fever over 101°F (38.3°C)

Difficulty swallowing or breathing

Dry socket (a painful, empty socket)

If you experience any of these symptoms, contact your dentist immediately.

Follow-Up Care

Follow up with your dentist as scheduled to remove stitches or check on the healing progress.

Take all prescribed medications as directed.

Emergency Contact Information

Dentist's name: _____________________________________

Dentist's phone number: _______________________________

After-hours emergency contact: _______________________

By following these instructions carefully, you can ensure a smooth and comfortable recovery after your tooth extraction. If you have any questions or concerns, don't hesitate to contact your dentist.

**2.Scenario 2:** Provide a clear, printable patient education handout detailing immediate steps for bystanders to take when a tooth is knocked out (avulsed), such as how to handle the tooth, options for placing it back in the socket or storing it, and the importance of seeking prompt dental care to maximize the chances of reimplantation.

**2.1. Chat GPT:**

What to Do if a Tooth is Knocked Out (Avulsed)

When a tooth is knocked out, quick action can increase the chances of saving the tooth. Follow the steps below to handle the situation properly and seek immediate dental care.

Immediate Steps to Take

If a tooth is knocked out, follow these steps as soon as possible:

• **Stay calm** and act quickly to improve the chances of saving the tooth.

• **Find the tooth** and pick it up by the crown (the chewing surface). Avoid touching the root of the tooth, as this can damage the delicate tissue that helps in reattachment.

Cleaning the Tooth

If the tooth is dirty, follow these guidelines for cleaning it:

• **Do not scrub the tooth** or use soap or chemicals to clean it.

• **Rinse the tooth gently** with milk or saline solution. If neither is available, use tap water. Make sure the water is clean and lukewarm.

Repositioning the Tooth

If possible, try to place the tooth back in its socket:

• **Hold the tooth by the crown** and carefully try to reposition it in the socket. Ensure it is oriented properly (the crown should face outward).

• Once the tooth is in place, **gently bite down on a clean cloth or gauze** to hold it in position.

• This step is especially important for **adult teeth**. If the knocked-out tooth is a baby tooth, do not attempt to reinsert it as it could damage the developing permanent tooth underneath.

If You Cannot Reinsert the Tooth

If you are unable to place the tooth back in the socket, it is crucial to keep it moist to preserve the root cells:

• Place the tooth in a **container of milk** or **saline solution**.

• If milk or saline is not available, place the tooth in the injured person's mouth between their **cheek and gums** (only if the person is conscious and able to hold the tooth safely).

• Avoid storing the tooth in **water**, as this can damage the root cells necessary for reimplantation.

• If none of these options are available, wrap the tooth in a clean cloth soaked with saliva or a small amount of water until you get to a dentist.

Seek Immediate Dental Care

Time is critical when a tooth is avulsed. The sooner the tooth is reinserted, the greater the chance of saving it:

• Contact a dentist or visit an **emergency dental clinic immediately**. Ideally, the tooth should be reimplanted within **30 minutes to 1 hour** for the best chance of success.

• If professional care is delayed, follow the steps above to keep the tooth moist and protected while on your way to the dentist.

Important Notes

• **Do not delay** seeking professional care. Even if the tooth is reinserted, the dentist will need to ensure it is properly aligned and stabilized.

• **Baby teeth** should not be reinserted, as this can damage the permanent teeth developing underneath.

If you have any concerns or need immediate assistance, contact your dentist:

Phone: [Insert Phone Number]

Email: [Insert Email Address]

Office Hours: [Insert Hours]

Quick action can save a knocked-out tooth. Follow these steps and seek dental care immediately to maximize the chances of successful reimplantation.

**1.2. Claude 3.5. Sonnet**

EMERGENCY: Knocked-Out Tooth Care Guide

TIME IS CRITICAL!

A knocked-out tooth has the best chance of survival if it's put back in place within 30 minutes.

1 Find the Tooth

Pick up the tooth by the crown (white part)

DO NOT touch the root (yellow part)

If dirty, gently rinse with milk or saline solution

DO NOT use water to clean the tooth

DO NOT scrub or dry the tooth

Try to Reinsert the Tooth

If the injured person is conscious and able:

Gently place tooth back in socket

Ensure it's facing the right way

Bite down gently on gauze or clean cloth

3 If You Cannot Reinsert the Tooth

Store the tooth in order of preference:

Milk (cold and fresh)

Saline solution

Save-A-Tooth solution (if available)

Between cheek and gum (if patient is conscious and old enough)

DO NOT store in water or let the tooth dry out!

 Seek Immediate Dental Care

Go to an emergency dentist immediately

If after hours, visit an emergency room

Bring the tooth in its storage medium

Additional Tips:

Apply cold compress to face for pain/swelling

Control bleeding with clean gauze

Take over-the-counter pain medication if needed

Emergency Dental Contacts:

Dentist: (XXX) XXX-XXXX
Emergency After Hours: (XXX) XXX-XXXX
Emergency Room: (XXX) XXX-XXXX

**1.3. Gemini 1.5. Flash**

Immediate Actions for a Knocked-Out Tooth (Avulsion)

This information is for emergency situations only. Seek immediate professional dental care.

A knocked-out tooth is a dental emergency requiring immediate action. The faster you act, the greater the chance of saving the tooth. Follow these steps carefully:

1. Find the Tooth:

Locate the tooth as quickly as possible. Handle the tooth by the crown (the white part), avoiding touching the root (the part that was in the gum).

2. Handle the Tooth Carefully:

Do NOT: Clean the tooth with soap, water, or any other cleaning agents. Do not scrub or wipe the tooth.

Do: Gently rinse the tooth with milk or saliva if it is visibly dirty. Avoid harsh scrubbing.

3. Reimplantation (Putting the Tooth Back In):

Ideal Scenario: If possible, and you are comfortable doing so, gently attempt to reinsert the tooth back into its socket. Try to align it as accurately as possible.

Hold the Tooth in Place: If reimplanted, gently bite down on a clean gauze pad or cloth to hold the tooth in place.

4. Storage (If Reimplantation is Not Possible):

Best Option: If you cannot reimplant the tooth, place it in a container of milk, saliva (in an adult's mouth), or a commercially available tooth preservation solution (e.g., Save-a-Tooth). Milk is the best readily available option.

Acceptable Options: If milk or saliva are unavailable, use a clean container filled with saline solution (salt water). Avoid using water alone.

Keep it Moist: The most crucial factor is to keep the tooth moist at all times. A dry tooth will not survive.

5. Transport to the Dentist:

Time is Critical: Bring the tooth and the patient to a dentist or emergency room immediately. The sooner the tooth is treated, the better the chances of successful reimplantation.

Transport Safely: Keep the tooth in its storage container and transport it carefully.

6. What to Tell the Dentist:

Be prepared to tell the dentist:

How the injury occurred.

How long the tooth has been out of the socket.

How the tooth was handled and stored.

Important Considerations:

Children: If the injured person is a child, remain calm and reassure them. Follow the above steps as quickly as possible.

Adult Supervision: Adult supervision is crucial, especially with children.

Other Injuries: Check for other injuries. If there is significant bleeding or other injuries, seek immediate medical attention.

This information is for emergency situations only. It does not replace professional dental advice. Seek immediate dental care to maximize the chances of saving the avulsed tooth.

Contact Information:

[Emergency Dental Contact Information]

[Local Emergency Room Contact Information]

Disclaimer: This handout provides general guidance for a dental emergency. Individual situations may vary. This information is not a substitute for professional medical advice.

**4.1. Llama 3.1-405b**

Emergency Steps for a Knocked-Out Tooth (Avulsed Tooth)

Act Fast to Save the Tooth!

If a tooth is knocked out, it's essential to act quickly and carefully to increase the chances of successful reimplantation. Follow these steps:

Step 1: Handle the Tooth with Care

Pick up the tooth by the crown (the part that's visible in the mouth), not the root.

Avoid touching the root or any attached tissue.

Gently rinse the tooth with cold water to remove any dirt or debris.

Step 2: Reinsert the Tooth (if possible)

If the tooth is clean and undamaged, try to reinsert it into the socket.

Make sure the tooth is facing the correct direction (the crown should be facing upwards).

Gently push the tooth into the socket until it clicks into place.

Bite on a clean cloth or gauze to hold the tooth in place.

Step 3: Store the Tooth (if reinsertion is not possible)

If the tooth cannot be reinserted, store it in one of the following:

Cold milk

Cold water with a pinch of salt

A commercial tooth preservation kit (available at pharmacies)

A clean cloth or gauze soaked in cold water

Avoid storing the tooth in:

Hot water

Tap water

The mouth (unless it's reinserted into the socket)

Step 4: Seek Prompt Dental Care

See a dentist or visit an emergency room within 30-60 minutes of the injury.

The sooner the tooth is treated, the higher the chances of successful reimplantation.

Even if the tooth is reinserted, it's crucial to have it evaluated by a dentist to ensure proper healing and prevent infection.

Additional Tips

Apply a cold compress to the affected area to reduce swelling.

Take over-the-counter pain relievers, such as ibuprofen (Advil, Motrin) or acetaminophen (Tylenol), as directed.

Avoid biting or chewing on the affected area until the tooth is evaluated by a dentist.

Remember

A knocked-out tooth is a dental emergency that requires prompt attention.

Acting quickly and following these steps can increase the chances of successful reimplantation.

If you're unsure about what to do, contact a dentist or visit an emergency room for guidance.

Emergency Contact Information

Dentist's name: _____________________________________

Dentist's phone number: _______________________________

After-hours emergency contact: _______________________

**3.Scenario 3:** Provide a clear, printable patient education handout that explains the proper daily tooth brushing technique to maintain optimal oral hygiene. This should include step-by-step instructions on brushing angles, pressure, duration, and tips on choosing the right toothbrush and toothpaste for effective plaque removal.

**2.1. Chat GPT:**

Proper Tooth Brushing Technique

Brushing your teeth correctly is essential for maintaining good oral hygiene and preventing problems like cavities, gum disease, and bad breath. Follow these step-by-step instructions to ensure you are cleaning your teeth effectively.

Step-by-Step Brushing Instructions

To properly brush your teeth, follow these steps:

1. Use the Right Toothbrush

- Choose a toothbrush with **soft bristles** to prevent damage to your gums and tooth enamel.
- The toothbrush head should be small enough to reach all areas of your mouth, including the back teeth.
- Consider using an **electric toothbrush**, which can be more effective at removing plaque, especially if you have difficulty brushing manually.

2. Use Fluoride Toothpaste

- Choose a **fluoride toothpaste** to help strengthen your teeth and prevent cavities.
- Make sure the toothpaste has the **ADA Seal of Approval**, indicating that it meets safety and effectiveness standards.

3. Place the Toothbrush at a 45-Degree Angle

- Hold your toothbrush at a **45-degree angle** to your gums. This allows the bristles to clean under the gumline where plaque and bacteria accumulate.
- Brush gently in **small circular motions**, focusing on one or two teeth at a time.

4. Brush All Surfaces of the Teeth

- Brush the **outer surfaces** of your teeth, starting from the back and moving toward the front.
- Next, brush the **inner surfaces** of your teeth using the same small circular motions.
- Brush the **chewing surfaces** of your teeth using a back-and-forth motion.

5. Brush for a Full Two Minutes

- Make sure to brush for at least **two minutes** to ensure all areas of your mouth are thoroughly cleaned.
- To help keep track of time, you can use a **timer** or listen to a two-minute song while brushing.

6. Be Gentle with Your Gums

- When brushing near your gums, use gentle pressure. **Do not scrub forcefully**, as this can cause gum recession and wear down your tooth enamel.

7. Brush Your Tongue

- Gently brush your tongue or use a **tongue scraper** to remove bacteria and freshen your breath.
- Start from the back of your tongue and move toward the tip, using light pressure.

8. Rinse Your Mouth and Toothbrush

- After brushing, **rinse your mouth** with water or mouthwash to remove any remaining toothpaste or debris.
- **Rinse your toothbrush** thoroughly and store it in an upright position, allowing it to air dry.

Tips for Effective Brushing

- **Brush twice a day**: Brush your teeth in the morning and before bed to remove plaque and prevent tartar buildup.
- **Replace your toothbrush** every 3 to 4 months, or sooner if the bristles become frayed. A worn-out toothbrush is less effective at cleaning your teeth.
- If you have **sensitive teeth or gums**, talk to your dentist about using a toothbrush designed for sensitivity and a desensitizing toothpaste.
- After brushing, consider using **floss** to clean between your teeth where your toothbrush cannot reach.

Signs You May Be Brushing Incorrectly

If you notice any of the following signs, you may need to adjust your brushing technique:

- Bleeding gums after brushing, which could indicate that you are brushing too hard or have gum disease.
- Receding gums or tooth sensitivity, which may result from excessive pressure or using a hard-bristled toothbrush.
- Plaque buildup or bad breath even after brushing, which could mean you are not brushing long enough or missing certain areas.

If you have any concerns about your brushing technique or oral health, contact your dentist for advice:

Phone: [Insert Phone Number]

Email: [Insert Email Address]

Office Hours: [Insert Hours]

Proper brushing is essential to maintaining healthy teeth and gums. Follow these steps to ensure you're cleaning your teeth effectively and preventing oral health problems.

**1.2. Claude 3.5. Sonnet**

Proper Tooth Brushing Technique Guide

Essential Tools

Selecting Your Toothbrush:

- Soft or extra-soft bristles
- Small head for better access
- Comfortable grip handle
- Replace every 3-4 months

Choosing Toothpaste:

- Look for fluoride content
- ADA (American Dental Association) approved
- Consider specific needs (sensitivity, whitening)

Step-by-Step Brushing Technique

1. Preparation

- Use a pea-sized amount of toothpaste
- Wet toothbrush slightly
- Hold brush at 45-degree angle to gums

2. Outer Surfaces

- Use gentle circular motions
- Brush 2-3 teeth at a time
- Maintain 45-degree angle

3. Inner Surfaces

- Tilt brush vertically for front teeth
- Use up-and-down strokes
- Maintain gentle pressure

4. Chewing Surfaces

- Use back-and-forth strokes
- Clean all grooves thoroughly

5. Tongue and Roof

- Gentle strokes on tongue
- Clean roof of mouth

Important Tips

- Duration: Brush for at least 2 minutes
- Frequency: Brush at least twice daily
- Pressure: Use light pressure - just enough to feel bristles
- Timing: Always brush before bed and after breakfast
- Order: Follow the same pattern each time to ensure no areas are missed

Common Brushing Mistakes to Avoid:

- Brushing too hard
- Rushing through brushing
- Using worn-out brushes
- Brushing immediately after acidic foods
- Missing the gum line
- Rinsing immediately after brushing

Remember: Proper brushing technique combined with daily flossing and regular dental check-ups is key to maintaining optimal oral health.

For questions or concerns, contact your dental office:
Phone: (XXX) XXX-XXXX

**1.3. Gemini 1.5. Flash**

Mastering the Art of Toothbrushing: A Guide to Optimal Oral Hygiene

This handout provides a step-by-step guide to effective toothbrushing. Following these instructions will help you maintain optimal oral health and prevent dental problems.

I. Choosing the Right Tools:

Toothbrush: Select a soft-bristled toothbrush with a head small enough to reach all areas of your mouth comfortably. Consider an electric toothbrush; studies show they can be more effective at plaque removal than manual toothbrushes. Replace your toothbrush every 3-4 months, or sooner if the bristles become frayed.

Toothpaste: Choose a fluoride-containing toothpaste. Fluoride strengthens tooth enamel and helps prevent cavities. Consider a toothpaste that also contains ingredients to help reduce sensitivity or whiten teeth if needed.

II. The Brushing Technique (Manual Toothbrush):

Angle: Hold your toothbrush at a 45-degree angle to your gums. This angle allows the bristles to reach under the gumline where plaque often accumulates.

Gentle Pressure: Use gentle, short strokes. Avoid scrubbing aggressively, as this can damage your gums and enamel. Think of "massaging" your teeth and gums.

Systematic Approach: Divide your mouth into sections to ensure thorough cleaning. Brush each section for about 5 seconds:

Outer Surfaces: Brush the outer surfaces of each tooth, moving from the gumline towards the biting edge.

Inner Surfaces: Brush the inner surfaces of each tooth, using short, gentle strokes. Pay special attention to the inner surfaces of your lower front teeth, where plaque often builds up.

Chewing Surfaces: Brush the chewing surfaces of your teeth using back-and-forth strokes.

Tongue: Gently brush your tongue from back to front to remove bacteria and freshen breath.

Duration: Brush for a full two minutes, twice a day (morning and evening). Use a timer or a toothbrush with a built-in timer to ensure you brush for the recommended time.

III. The Brushing Technique (Electric Toothbrush):

Angle: Hold the electric toothbrush at a 45-degree angle to your gums, as with a manual toothbrush.

Gentle Pressure: Let the electric toothbrush do the work. Avoid pressing too hard; the brush head should gently move across your teeth.

Systematic Approach: Follow the same systematic approach as with a manual toothbrush, ensuring you cover all surfaces of each tooth.

Duration: Most electric toothbrushes have a built-in timer that guides you to brush for the recommended two minutes.

IV. Additional Tips for Optimal Oral Hygiene:

Flossing: Floss daily to remove plaque and food particles from between your teeth and under the gumline where your toothbrush can't reach.

Mouthwash: Consider using a fluoride mouthwash to further enhance plaque control and freshen breath.

Regular Dental Checkups: Schedule regular checkups and cleanings with your dentist. Professional cleanings remove plaque and tartar that brushing and flossing may miss.

Diet: Maintain a healthy diet and limit sugary drinks and snacks.

V. Signs of Improper Brushing:

Bleeding gums: This may indicate gum disease.

Sensitivity: This may indicate worn enamel or exposed dentin.

Persistent bad breath: This may indicate poor oral hygiene.

If you experience any of these issues, consult your dentist.

Disclaimer: This handout provides general guidance on proper tooth brushing techniques. Individual needs may vary. This information is not a substitute for professional dental advice. Always consult your dentist for personalized recommendations.

**4.1. Llama 3.1-405b**

Proper Daily Tooth Brushing Technique

Maintaining optimal oral hygiene is crucial for a healthy smile. Brushing your teeth correctly is the first step towards a lifetime of good oral health. Follow these simple steps to ensure you're brushing your teeth like a pro!

Step 1: Choose the Right Toothbrush

Select a soft-bristled toothbrush that fits comfortably in your mouth.

Consider using an electric toothbrush, which can be more effective at removing plaque and improving gum health.

Replace your toothbrush every 3-4 months or sooner if the bristles become frayed.

Step 2: Select the Right Toothpaste

Choose a fluoride toothpaste that carries the American Dental Association (ADA) Seal of Acceptance.

For children under 3 years old, use a smear of toothpaste about the size of a grain of rice.

For children 3-6 years old, use a pea-sized amount of toothpaste.

For adults, use a pea-sized amount of toothpaste.

Step 3: Brush at the Right Angle

Place the toothbrush against your teeth at a 45-degree angle, pointing towards the gum line.

Gently brush the front, back, and top surfaces of your teeth.

Step 4: Brush with the Right Pressure

Use gentle circular motions to brush your teeth. Avoid using too much pressure, which can damage your gums.

Use a gentle touch, especially around the gum line.

Step 5: Brush for the Right Amount of Time

Brush your teeth for at least 2 minutes, twice a day.

Use a timer or a song to help keep track of time.

Step 6: Don't Forget the Often-Missed Areas

Brush the surfaces of your molars and the back sides of your teeth.

Don't forget to brush your tongue and the roof of your mouth to remove bacteria and freshen your breath.

Additional Tips

Brush your teeth at least 30 minutes after meals to allow the acid in your mouth to neutralize.

Avoid brushing your teeth too hard, which can damage your gums and tooth enamel.

Use a tongue scraper to remove bacteria and debris from the surface of your tongue.

Visit your dentist regularly for check-ups and cleanings to maintain optimal oral health.

Proper Brushing Technique Checklist

Use a soft-bristled toothbrush that fits comfortably in your mouth.

Choose a fluoride toothpaste with the ADA Seal of Acceptance.

Brush at a 45-degree angle, pointing towards the gum line.

Use gentle circular motions and avoid using too much pressure.

Brush for at least 2 minutes, twice a day.

Don't forget to brush often-missed areas, such as the surfaces of your molars and the back sides of your teeth.

By following these simple steps and tips, you'll be well on your way to maintaining optimal oral hygiene and a healthy, happy smile!

**4.Scenario 4:** Provide a clear, printable patient education handout outlining the steps for self-examination in oral cancer screening. Include guidance on how to visually and manually examine areas of the mouth, lips, and throat, what signs and symptoms to watch for (such as lumps, sores, or discoloration), and when to seek a dental professional's evaluation.

**2.1. Chat GPT**

Oral Cancer Self-Examination Guide

Oral cancer can develop in any part of the mouth, including the lips, tongue, gums, cheeks, roof of the mouth, and throat. Performing regular self-examinations can help detect early signs of oral cancer, increasing the chances of successful treatment. Follow the steps below to perform a thorough self-examination.

How to Perform a Self-Examination

You will need a bright light, a mirror, and clean hands. Follow these steps to examine different areas of your mouth:

1. Lips

- Stand in front of a mirror and pull your lips outward.
- Check for any sores, lumps, discoloration, or changes in texture.
- Run your fingers along the inside and outside of your lips to feel for any unusual bumps or areas of tenderness.

2. Gums

- Use a mirror to inspect the gums around your teeth.
- Look for any swelling, bleeding, or color changes (such as white, red, or dark patches).
- Use your fingers to gently feel along the gums for lumps or areas of sensitivity.

3. Inside of Cheeks

- Pull your cheeks away from your teeth using clean fingers.
- Examine the inner surface of your cheeks for red or white patches, sores, or lumps.
- Gently press your fingers along the inside of your cheeks to feel for any unusual growths.

4. Tongue

- Stick out your tongue and use a mirror to check for any discoloration, sores, or unusual textures on the top, sides, and underside of your tongue.
- Grasp your tongue with a clean cloth or gauze and gently pull it to one side, then the other, to inspect the sides more closely.
- Use your fingers to feel for any lumps or hard spots on your tongue.

5. Floor of the Mouth

- Lift your tongue and use a mirror to inspect the floor of your mouth.
- Look for any discoloration, sores, or swelling in this area.
- Use your fingers to gently feel for lumps or tender areas under your tongue.

6. Roof of the Mouth (Palate)

- Tilt your head back and use a mirror to examine the roof of your mouth.
- Check for any changes in color, sores, or unusual growths.
- Run your fingers along the roof of your mouth to feel for any unusual bumps or areas of tenderness.

7. Neck and Throat

- Use your fingers to gently feel around your neck and under your jaw for any lumps or swelling.
- Look at the back of your throat using a mirror. Check for any red or white patches, sores, or growths.
- Swallow a few times to ensure there is no pain or difficulty when swallowing.

What to Look For

During self-examination, be on the lookout for the following signs and symptoms of oral cancer:

- Red, white, or dark patches anywhere in your mouth or on your lips.
- Sores that do not heal within two weeks.
- Lumps or thickened areas in the cheeks, gums, or any other area of the mouth.
- Pain or difficulty swallowing or feeling like something is caught in your throat.
- Numbness or loss of sensation in any part of the mouth.
- Persistent sore throat or hoarseness.
- Unexplained bleeding from the mouth or gums.
- Loose teeth without an obvious cause like injury or gum disease.
- Jaw pain or stiffness that doesn't go away.

When to Seek Professional Evaluation

If you notice any of the following signs, contact your dentist or healthcare provider for a professional evaluation:

- Sores or patches that do not heal within two weeks.
- Unexplained lumps, growths, or bumps in your mouth, on your lips, or in your neck.
- Persistent pain, numbness, or difficulty swallowing.
- Any other unusual symptoms that last more than two weeks.

If you notice any unusual changes or have concerns, it is important to schedule an appointment with your dentist:

Phone: [Insert Phone Number]

Email: [Insert Email Address]

Office Hours: [Insert Hours]

Early detection is key in successfully treating oral cancer. Regular self-examinations, along with routine dental check-ups, can help detect any issues before they become serious.

**1.2. Claude 3.5. Sonnet**

Oral Cancer Self-Examination Guide

Important Information

• Perform this self-examination monthly

• Use a bright light and mirror

• You'll need: small mirror, gauze pad, bright light

• Best done after brushing teeth

1 Face and Neck Examination

• Look for swelling or asymmetry

• Feel both sides of neck for lumps

• Check for enlarged lymph nodes

• Note any skin changes or discoloration

2.Lips and Front of Mouth

• Examine lips (both outer and inner surfaces)

• Pull upper lip up and lower lip down

• Check color, texture, and any changes

• Look for sores or color changes

3 Cheek Examination

• Pull each cheek out to see inside surfaces

• Look for red or white patches

• Feel for lumps or tenderness

• Check both sides for symmetry

4 Tongue Inspection

• Stick out tongue, examine top surface

• Pull tongue side to side

• Use gauze to hold tip, check underneath

• Feel for lumps or rough spots

5 Floor of Mouth and Palate

• Touch roof of mouth with tongue

• Look up with mouth open wide

• Feel under tongue

• Check for changes in color or texture

Warning Signs to Watch For

• Sores that don't heal within 2 weeks

• White or red patches

• Lumps or thickened areas

• Difficulty swallowing or chewing

• Numbness in mouth or face

• Persistent sore throat

• Hoarseness lasting over 6 weeks

• Sensation of something caught in throat

Risk Factors

• Tobacco use (any form)

• Heavy alcohol consumption

• Excessive sun exposure

• Family history of cancer

• HPV infection

• Poor diet

• Age over 40

Seek Professional Evaluation If You Notice:

• Any changes lasting more than 2 weeks

• Unexplained bleeding

• Persistent pain

• Difficulty with normal functions

Contact Information:

Dentist: (XXX) XXX-XXXX

Emergency: (XXX) XXX-XXXX

Remember: Early detection significantly improves treatment outcomes.

**1.3. Gemini 1.5. Flash**

Oral Cancer Self-Examination: A Guide to Early Detection

Early detection of oral cancer significantly improves treatment outcomes and survival rates. Regular self-examinations are a crucial part of preventative oral healthcare. This handout provides a step-by-step guide to performing a thorough self-examination. Remember, this is not a substitute for professional dental care.

I. What to Look For:

Oral cancer can manifest in various ways. Be vigilant for any of the following signs and symptoms:

Sores or ulcers: Persistent sores or ulcers that don't heal within two weeks.

Lumps or thickenings: Unusual lumps, bumps, or thickenings in the mouth, lips, or neck.

White or red patches: White (leukoplakia) or red (erythroplakia) patches or areas of discoloration on the gums, tongue, or lining of the mouth.

Bleeding: Unexplained bleeding in the mouth.

Numbness or tingling: Numbness, tingling, or loss of feeling in any part of the mouth.

Difficulty chewing or swallowing: Persistent difficulty chewing, swallowing, or moving the jaw or tongue.

Loose teeth: Teeth that become loose without apparent reason.

Changes in voice: Hoarseness or changes in voice quality that persist.

Persistent sore throat: A sore throat that doesn't improve.

Ear pain: Persistent pain in the ear.

II. Performing the Self-Examination:

This examination should be performed in front of a mirror in good lighting. Use a tongue depressor or a clean finger to help visualize areas that are difficult to see.

Lips: Carefully examine your lips, both inside and outside, for any sores, lumps, discoloration, or unusual changes in texture.

Tongue: Inspect the top, bottom, and sides of your tongue. Gently pull your tongue out and examine its surface for any abnormalities. Pay close attention to the sides and the base of your tongue, where cancers often develop.

Cheeks: Gently pull back your cheeks to examine the inner lining of your cheeks. Look for any sores, lumps, or discolorations.

Gums: Inspect your gums carefully, looking for any unusual changes in color, texture, or the presence of sores or lumps.

Roof of the Mouth (Palate): Examine the roof of your mouth, including the hard and soft palates, for any abnormalities.

Floor of the Mouth: Lift your tongue to examine the floor of your mouth. Feel for any lumps or thickenings.

Throat (as far as you can see): Open your mouth wide and look into your throat as far as you can comfortably see. Check for any abnormalities.

Neck: Feel your neck for any swollen lymph nodes (glands). Lymph nodes are small, bean-shaped glands that are usually not palpable. If you feel any enlarged or tender lymph nodes, it's important to have them checked.

III. Frequency of Self-Examination:

Perform a thorough self-examination at least once a month. Familiarize yourself with the normal appearance of your mouth so you can easily detect any changes.

IV. When to Seek Professional Evaluation:

If you notice any of the warning signs listed above, or if you have any concerns about the appearance of your mouth, contact your dentist or doctor immediately. Early detection is critical for successful treatment.

V. Professional Dental Care:

Regular dental checkups and cleanings are essential for early detection of oral cancer. Your dentist can perform a thorough examination and identify any potential problems.

Disclaimer: This handout provides general guidance on self-examination for oral cancer. It is not a substitute for professional medical advice. Early detection is crucial; consult your dentist or doctor immediately if you have any concerns.

**4.1. Llama 3.1-405b**

Oral Cancer Self-Examination Guide

Early detection is key in the fight against oral cancer. By performing a monthly self-examination, you can help identify potential issues and seek professional evaluation if necessary. Follow these steps to examine your mouth, lips, and throat for signs of oral cancer.

Step 1: Prepare for the Examination

Stand in front of a mirror with good lighting.

Remove any dentures, oral appliances, or piercings.

Rinse your mouth with water to remove any debris.

Step 2: Examine Your Lips

Look for any changes in the color, texture, or shape of your lips.

Check for any lumps, bumps, or sores.

Gently pull your lower lip down and examine the inside of your lip.

Repeat this process for your upper lip.

Step 3: Examine Your Mouth

Open your mouth wide and examine the inside of your cheeks.

Look for any red or white patches, lumps, or sores.

Check the roof of your mouth, including the soft palate and the uvula.

Examine your tongue, including the top, bottom, and sides.

Gently lift your tongue and examine the underside.

Step 4: Examine Your Gums and Teeth

Look for any changes in the color or texture of your gums.

Check for any lumps, bumps, or sores on your gums or teeth.

Examine the areas where your teeth and gums meet.

Step 5: Examine Your Throat

Look for any changes in the color or texture of your throat.

Check for any lumps, bumps, or sores on your tonsils or the back of your throat.

Gently tilt your head back and examine the roof of your throat.

Signs and Symptoms to Watch For

Lumps, bumps, or sores that do not heal within 2 weeks

Red or white patches on the lips, tongue, or inside of the mouth

Difficulty swallowing or speaking

Pain or numbness in the mouth, lips, or throat

Changes in the color or texture of the mouth, lips, or throat

Unexplained bleeding or swelling

When to Seek a Dental Professional's Evaluation

If you notice any of the signs or symptoms listed above.

If you have a history of tobacco or alcohol use.

If you have a history of oral cancer or other cancers.

If you have a weakened immune system.

If you are experiencing persistent pain or discomfort in your mouth, lips, or throat.

Remember

Early detection is key in the fight against oral cancer.

Perform a monthly self-examination to identify potential issues.

Seek a dental professional's evaluation if you notice any signs or symptoms.

A thorough examination by a dental professional can help detect oral cancer in its early stages.

By following these steps and being aware of the signs and symptoms of oral cancer, you can help protect your health and well-being. If you have any concerns or questions, don't hesitate to consult with your dental professional.

**Supplemental File 2: Fleiss Kappa values for inter rater reliability**

| **Source** | **Scenario** | **Fleiss kappa value*** | **CI Upper limit** | **CI Low limit** | **Agreement level** |
| --- | --- | --- | --- | --- | --- |
| Chat GPT 4.o | 1 | 0.69 | 0.81 | 0.56 | Substantial |
|  | 2 | 0.52 | 0.65 | 0.39 | Moderate |
|  | 3 | 0.57 | 0.69 | 0.44 | Moderate |
|  | 4 | 0.53 | 0.65 | 0.40 | Moderate |
| Claude 3.5 Sonnet | 1 | 0.45 | 0.58 | 0.32 | Moderate |
|  | 2 | 0.58 | 0.70 | 0.45 | Moderate |
|  | 3 | 0.66 | 0.78 | 0.53 | Substantial |
|  | 4 | 0.56 | 0.44 | 0.69 | Substantial |
| Gemini 1.5 Flash | 1 | 0.73 | 0.60 | 0.85 | Substantial |
|  | 2 | 0.78 | 0.65 | 0.90 | Substantial |
|  | 3 | 0.78 | 0.65 | 0.90 | Substantial |
|  | 4 | 0.79 | 0.64 | 0.87 | Substantial |
| Llama 3.1-405b | 1 | 0.83 | 0.70 | 0.95 | almost perfect agreement |
|  | 2 | 0.82 | 0.69 | 0.94 | almost perfect agreement |
|  | 3 | 0.89 | 0.76 | 1.01 | almost perfect agreement |
|  | 4 | 0.78 | 0.65 | 0.90 | almost perfect agreement |

*values ≤ 0 as indicating no agreement; 0.01–0.20 as none to slight; 0.21–0.40 as fair; 0.41– 0.60 as moderate; 0.61–0.80 as substantial, and 0.81–1.00 as almost perfect agreement.
